# Activity and Characterization of Tocopherol Oxidase in Corn Germs and Its Relationship with Oil Color Reversion

**DOI:** 10.3390/molecules28062659

**Published:** 2023-03-15

**Authors:** Liyou Zheng, Miaomiao Zhu, Fei Zhang, Jun Jin, Qingzhe Jin, Hongyan Guo

**Affiliations:** 1 School of Biological and Food Engineering, Anhui Polytechnic University, Wuhu 241000, China; zhengliyou@ahpu.edu.cn (L.Z.);; 2 Collaborative Innovation Center of Food Safety and Quality Control in Jiangsu Province, National Engineering Research Center for Functional Food, School of Food Science and Technology, Jiangnan University, Wuxi 214122, China

**Keywords:** tocopherol oxidase, γ-tocopherol oxidation, tocored, properties, enzymatic oxidation

## Abstract

Color reversion has long been a major problem for the vegetable oil industry, and the enzymatic oxidation of γ-tocopherol is thought to trigger this phenomenon. In this study, first, the extraction, purification, and detailed characterization of tocopherol oxidase from fresh corn germs were performed. Then, the relationship between the enzyme reaction of γ-tocopherol and oil color reversion was verified. The results showed that the membrane-free extracts of raw corn germ performed specific catalysis of tocopherol in the presence of lecithin. In terms of the oxidation product, tocored (the precursor of color reversion) was detected in the mixture after the catalytic reactions, indicating that this anticipated enzyme reaction was probably correlated with the color reversion. Furthermore, the optimal pH and temperature for the tocopherol oxidase enzyme were 4.6 and 20 °C, respectively. In addition, ascorbic acid at 1.0 mM completely inhibited the enzymatic reaction.

## 1. Introduction

Under certain conditions, edible vegetable oils that are refined, bleached, and deodorized to yield a pale light-colored product become darker during storage [1,2]. This phenomenon, known as color reversion, has long been a problem for the vegetable oil industry [1,3,4]. It deteriorates oil color stability and affects oil appearance [2,4]. The degree of color change during storage may depend on raw seed quality, the moisture content of oilseeds, refining and deodorization conditions, storage conditions, temperature, exposure to light, and contact with air during storage, etc. [1,5]. Furthermore, it has been reported that the tendency of different oils to undergo color reversion is variable, with corn oil and soybean oil requiring only a few hours for the process to occur, whereas other oils require several months [1,5].

Given its notorious influence on the oil industry, oil color reversion has been extensively studied in the past few decades [6,7,8,9,10]. On the one hand, γ-tocopherol and its oxidant products (such as tocored) were thought to be involved in the mechanism of color reversion [1,2,3,4,9]. On the other hand, the moisture content of oilseeds also appears to be a key factor in promoting color reversion in fully refined oils [1,3,10]. It was then reported that the moisture of soybeans markedly affected the quantity of tocopherol in the crude oil and was closely related to color reversion [7]. That is, soybeans with high moisture (16–20%) tend to have less γ-tocopherol content, which means more γ-tocopherol would be consumed and change to other color-causing substances, thus color reversion probably occurs [3,11]. In addition, the nature of raw material (moisture contents, degree of crushing, and preheating temperatures) was found to be closely related to the content changes of γ-tocopherol [3,8]. Meanwhile, the temperatures applied during the pretreatment processes of oilseeds also dramatically influence γ-tocopherol content [3]. Furthermore, several studies have pointed out that the degradation of γ-tocopherol during the pretreatment of oil extraction was probably caused by enzymatic reactions [3,8,12,13]. Therefore, it would be of great significance to determine the presence of an enzyme that is able to catalyze the oxidation of tocopherol and discover the possible mechanism in the process of oil color reversion.

It is generally believed that the tocopherol degradation is most commonly ascribed to non-enzymatic oxidation [14], in which the tocopherol is directly catalyzed by reactive oxygen species due to its antioxidant function [13,15,16]. However, increasing evidence has been put forward to indicate that this phenomenon is involved enzymatic reactions. Though only limited information is available, tocopherol oxidase has been discussed continually by several researchers [12,13,17,18,19,20,21]. Tocopherol oxidase has been found in many plant organs, such as roots, stems, leaves, flowers, and fruits [18]. The properties of tocopherol oxidase were initially investigated in different plant varieties [18,19,22]. It is able to oxidize the four kinds of tocopherols with different catalytic efficiencies [18,22,23]. Furthermore, it is sensitive to elevated temperatures and different pH values [18,20,21]. Although little information exists on this oxidase in corn germs, all these results shed light on our investigations. Since the major tocopherol form is γ-tocopherol in corn germ oil [24], and tocopherol and its oxidation are also thought to be involved in the mechanism of corn oil color reversion [1,4], γ-tocopherol is often chosen as the enzyme-catalyzed substrate. Thus, the aims of the present study were to verify the occurrence of tocopherol oxidase activity in corn germ and further achieve preliminary purification. Additionally, detailed biochemical analysis (including substrate specificity, optimum pH, and temperature), and the chemical inhibitors were further investigated. Finally, the possible products of any enzymatic reaction were determined in order to understand the relationship between the enzyme and oil color reversion.

## 2. Results and Discussion

### 2.1. Verification of the Catalytic Activity of Cell-Free Extracts

The goal of this study was to explore the relationship between tocopherol oxidase and color reversion in corn oil, and sun-dried corn is usually used to produce corn germ for partial press and extraction by hexane in the oil industry [25]. Corn germs are a byproduct of starch processing. Here, corn germs were used to obtain the cell-free extracts containing crude enzymes. Generally, to our knowledge, extracts show the highest activity in fresh plant tissues. So, in this study, corn germs from two different sources were tested and compared to obtain cell-free extracts. Several studies extracted tocopherol oxidase from different plant varieties [18,19,22]. This current study was the first time that this oxidase was investigated in corn germs, so we applied the reported method with some adjustments to extract oxidase from the corn germ [18]. The obtained crude extract was stored at 4 °C for the following experiments.

**Figure 1:**
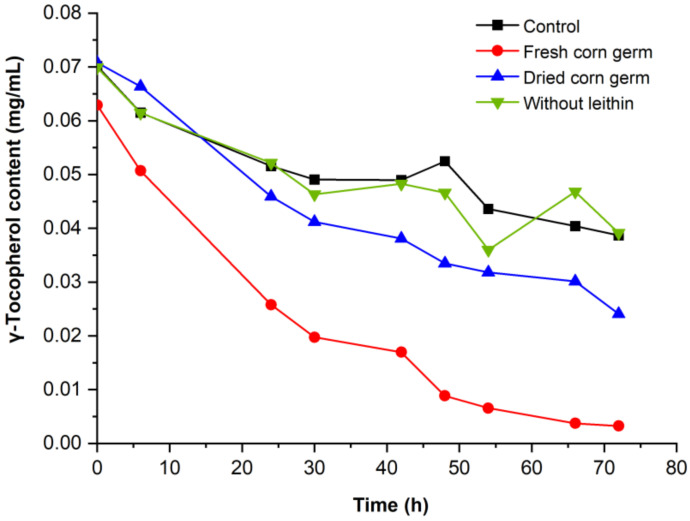
Residual γ-tocopherol content during the reaction (0–72 h).

Figure 1 shows the variation in γ-tocopherol content with reaction time. The γ-tocopherol content in all samples declined significantly (*p* < 0.05) with increasing reaction time. As can be seen from Figure 1, the γ-tocopherol content was significantly lower (*p* < 0.05) with respect to the control, indicating that the cell-free extract indeed possessed tocopherol catalytic activity. The majority of the γ-tocopherol (95%) was oxidized by the crude extract in the fresh corn germ, whereas, there was a 66% reduction in the dried corn germ. Obviously, fresh corn germs displayed higher enzyme activity than dried corn germs. Furthermore, γ-tocopherol was not oxidized by the cell-free homogenates in the absence of lecithin, proving that lecithin is required in the enzyme reaction processes, which is consistent with published research [18,22]. The emulsion containing lecithin was able to accelerate the contact between the oxidase and the substrate, which meant that tocopherol can be incorporated into phospholipid aggregates to trigger enzyme reactions.

**Figure 2:**
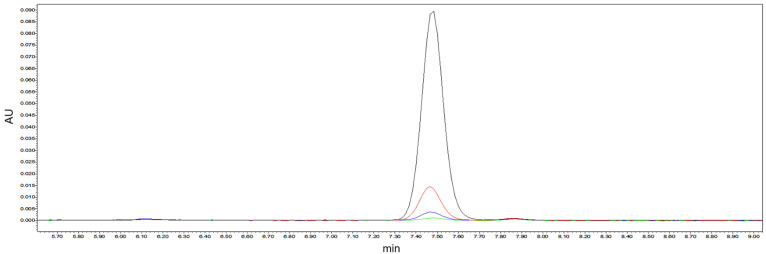
The chromatograms of γ-tocopherol content at the reaction times of 0 h (black line), 48 h (red line), 66 h (blue line), and 72 h (green line).

As shown in Figure 2, the amount of γ-tocopherol decreased significantly (*p* < 0.05) as the reaction proceeded, and the reaction time was found to be so long that it was hard to define the enzyme activity, unlike with most other enzymes [26]. Meanwhile, the extent of the enzyme reaction was indicated by changes in substrate content (γ-tocopherol) due to the unknown oxidation product. Therefore, the decrease in tocopherol content over a certain period of time was used to evaluate the enzyme activity in this study.

### 2.2. Different Activity towards Tocopherol Homologs

**Figure 3:**
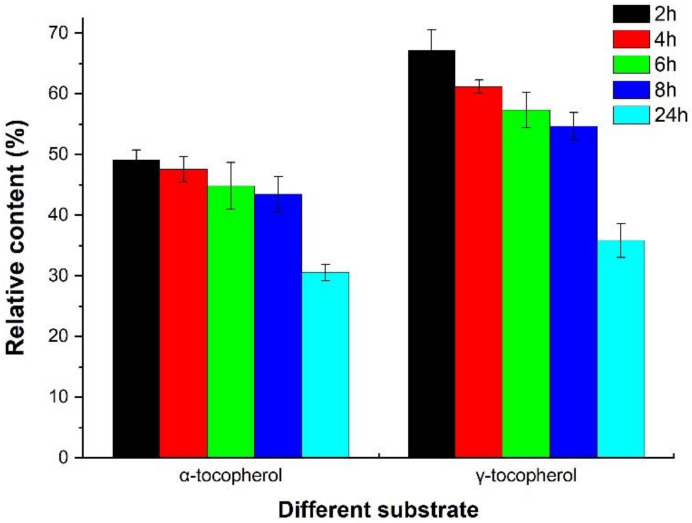
Comparison of the relative contents of α- and γ-tocopherols after different reaction times.

As α-tocopherol and γ-tocopherol are major tocopherol homologs in corn germ oil [24], the two tocopherols were chosen as the substrates to investigate oxidase activity in this study. For each tocopherol, the relative content decreased along with the duration. The results showed that significant differences in the activity of the oxidase between the α- and γ-tocopherols were observed (Figure 3), which indicated that the enzyme obtained from the corn germ has preferential selectivity for particular tocopherol homologs.

According to previous research, namely studies on the substrate specificity of oxidase, tocopherol oxidase is able to catalyze four different tocopherols [23]. Moreover, the results of the current paper are consistent with the results reported by Szymańska et al. [18], who found that the rate of the catalysis reaction oxidized by the extract from the etiolated shoots of *p. coccineus* was in the following order: α- > β- > γ- > δ-tocopherol. Meanwhile, the enzyme from *Pisum sativum* L. seeds is not specific to α-tocopherol but will also catalyze the oxidation of other tocopherol homologs at a lower rate. Activities can be ranked in the following order: α- > β- = γ- > δ-tocopherol [22]. Conversely, another study showed that fresh *P. sativum* extract presented higher activity against γ-tocopherol than α-tocopherol [23]. The differences among these results are probably due to the different plant species and the reaction conditions used. We will test oxidase activity against β- and δ-tocopherols in future works.

Interestingly, we unexpectedly observed that the reaction rates of different tocopherol homologs were dependent on the duration of the reaction. That is, the oxidase showed higher activity against α-tocopherol when the reaction time was under 24 h. Conversely, the degradation rate of γ-tocopherol was faster than that of α-tocopherol when the reaction time exceeded 24 h. This phenomenon had never been reported before, given that reaction time was limited in previous research. The involved mechanisms are unclear and warrant further exploration.

### 2.3. Analysis of the Oxidation Products of Tocopherol

**Figure 4:**
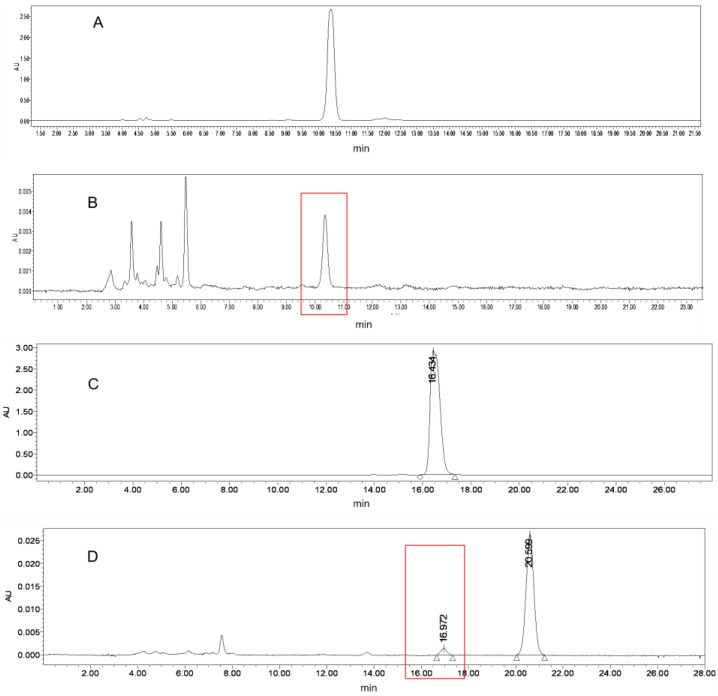
Analysis of reaction products ((**A**), γ-TQ standard in HPLC; (**B**), the peak of γ-TQ in the sample; (**C**), tocored standard in HPLC; (**D**), the peak of tocored in the sample).

The oxidation products of α-tocopherol and γ-tocopherol were analyzed (Figure 4). The results obtained from HPLC showed that α-TQ was the main product of α-tocopherol, which is in line with the results of a previous study [20]. As for γ-tocopherol, the oxidation products were more complicated. In our first experiment, γ-TQ was detected and considered as the primary product of γ-tocopherol, as is seen in α-tocopherol. However, we detected a new product when we reduced the reaction time, the content of which first increased and then decreased with time. Through comparison with the peak time of the standard substance, this compound was identified as tocored, the precursor for color reversion [27].

A possible explanation for this phenomenon is that tocored was formed during the early stages of the enzyme reaction and was converted to γ-TQ as the reaction time extended. The mechanism of this phenomenon is still unclear and should be further studied. The detection of tocored indicated a possible linkage between tocopherol enzyme oxidation and color reversion since tocored has been referred to as the precursor for color reversion by previous studies [1,4,10]. Given that tocored is considered to be a main precursor of color reversion [3] and that the enzyme reaction generated tocored in the present study, this means that color reversion can probably be accelerated by the enzymatic reaction that occurs in the pretreatment of raw oilseeds. For instance, the enzymes come into contact with the substrates as cell destruction takes place during flaking and crushing processes. In this respect, if the moisture and temperature are suitable for oxidase activity, γ-tocopherol can be oxidized and cause a significant loss of its content. Thus, the content of color reversion precursors is increased and color reversion occurred.

### 2.4. Characterization of the Oxidase

#### 2.4.1. Effects of pH and Temperature on the Tocopherol Oxidase Activity

The pH and temperature are two basic indices for enzymes. In this study, the effects of pH and temperature on the oxidase activity were examined. The activity of the oxidase was evaluated by the degradation of γ-tocopherol.

**Figure 5:**
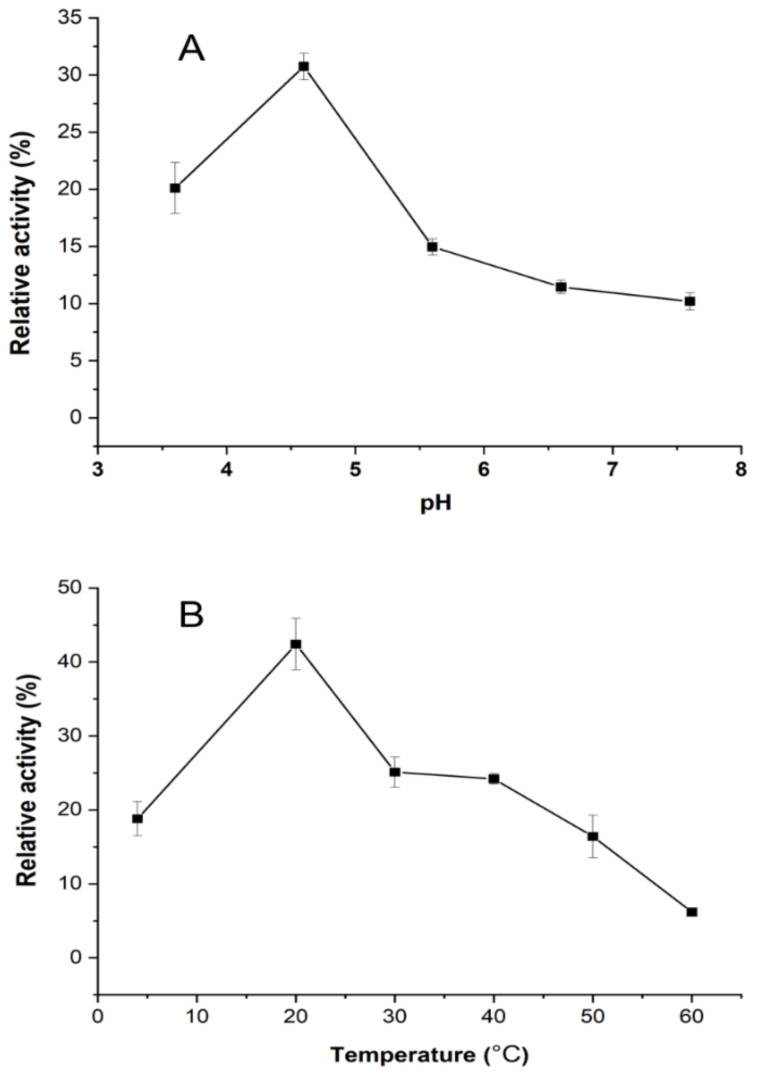
Effect of pH and temperature on the activity of tocopherol oxidase from fresh corn germs ((**A**), activity of tocopherol oxidase as a function of pH at room temperature; (**B**), activity of tocopherol oxidase as a function of temperature with optimal pH).

As shown in Figure 5A, the optimum pH for tocopherol oxidase’s catalysis of γ-tocopherol was 4.6. A pH optimum of 5.5 for the oxidase extracted from *Pisum sativum* L. shoots was reported [20,21,28]. The differences may arise from the different plant varieties applied to extract the tocopherol oxidase. Furthermore, the oxidase unexpectedly showed relatively high stability regarding extreme pH changes. The low activity of tocopherol oxidase in the acidic pH range can be exploited by adding certain acidic solutions to inhibit the enzymatic oxidation of tocopherol in raw material during storage and subsequent processing.

The effect of different temperatures on the activity is shown in Figure 5B. Both low (4 °C) and elevated temperatures (50 and 60 °C) demonstrated an inhibitory effect on the oxidase activity. The oxidase shows the highest catalysis activity at 20 °C and then dropped sharply by 41% when the temperature increased up to 30 °C. Similar findings concerning the activity of tocopherol oxidase in the fresh homogenates of the etiolated shoots of *Phaseolus coccineus* seedlings had previously been reported [18]. This information has practical significance for the storage of raw materials, as corn awaiting oil extraction is commonly stored at room temperature, close to the optimum temperature of the oxidase.

Although temperature optima are variety-dependent, tocopherol oxidase showed higher sensitivity to temperature than polyphenol oxidase (PPO) in this study.

#### 2.4.2. Effect of Inhibitors on Enzyme Activity

L-cysteine and ascorbic acid, which are the two main chemical inhibitors of PPO [29], were added to the reaction systems in order to assess their inhibitory effects on tocopherol oxidase using γ-tocopherol as substrate at optimal conditions. The results are expressed as inhibition rates as shown in Table 1. During the test, ascorbic acid had significantly stronger inhibitive effects on the reaction than L-cysteine, while both substances demonstrated significant differences compared to the control (*p* < 0.05). Ascorbic acid, as a common antioxidant, is able to reduce quinones and has been shown to irreversibly inhibit PPO [30]. It also displayed a complete inhibition of tocopherol oxidase, and similar effects have been reported [28]. However, the mechanism was different from the PPO, as in the previous study, the removal of the ascorbate recovered activity [18], indicating that ascorbic acid is not a direct inhibitor of the enzyme. L-cysteine was used as an effective alternative to other PPO inhibitors in certain food applications. In the present study, cysteine partially inhibited oxidase activity. It is likely that cysteine (1 mM) was also used to evaluate its inhibitory effect on the activity of the fresh homogenates of the etiolated shoots of *P. coccineus*. After 60 min, 42% α-tocopherol oxidation occurred, while 84% α-tocopherol oxidation occurred in the control group, which meant that half of the oxidase activity was inhibited by cysteine [18]. In addition, the reaction system was completely inhibited by cyanide and ascorbic acid. Alongside these two inhibitors, sulphite, EDTA [18,28], and azide [18] were also effective inhibitors and are traditionally used during crushing and processing, which will be tested in a later study.

## 3. Materials and Methods

### 3.1. Reagents and Materials

Standards of α-, β-, γ-, and δ-tocopherols (purity > 95%) were purchased from Sigma-Aldrich Chemical Co. Ltd. (Shanghai, China). n-Hexane, methanol, and 2-propanol of chromatography grade were obtained from J&K Chemicals (Shanghai, China). Unless otherwise stated, all other chemicals and reagents (e.g., sodium citrate, sodium dihydrogen phosphate) were of analytical grade and provided by Sinopharm Chemical Regent (Shanghai, China).

### 3.2. Plant Material and Enzyme Extraction

The fresh corn used in this study was purchased from a local market in the region of Wuxi, Jiangsu Province, China (from July 2018 through November 2018). Firstly, the corn germs were peeled off from the corn with tweezers and then stored at 4 °C until used for the enzyme extraction. The cell-free homogenate extraction was performed according to the procedures reported previously [18] with some modifications. Briefly, the fresh germ was homogenized for 60 s using a blender (Philips, HR2006, Zhuhai, China) in 50 mM citrate–phosphate buffer (pH 5.5) with a ratio of 8 mL buffer per gram of germ. Then, the homogenate was filtered through a strainer with a 300 mesh to remove residues, followed by centrifugation with a high-speed freezing centrifuge (Eppendorf AG, 22,331 Hamburg, 5811EL375466, Hamburg, Germany) at 10,000× *g* for 25 min at 4 °C. Then, the supernatant was stored at 4 °C for the later extraction of crude enzyme.

### 3.3. Partial Purification

**Figure 6:**
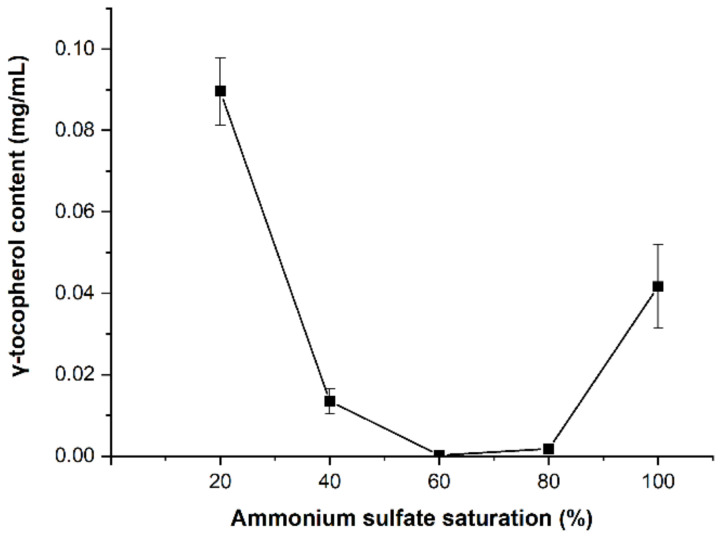
Content of residue γ-tocopherol after reaction with the crude enzyme obtained by ammonium sulfate precipitation.

In this study, the preliminary purification of the oxidase was performed. First, the cell-free homogenate obtained from the extraction was subject to ammonium sulfate precipitation within a wide range of 20–80%. The profile of ammonium sulfate precipitation is given in Figure 6. The content of γ-tocopherol decreased dramatically as the ammonium sulfate saturation increased from 20% to 60%. The results indicated that the enzyme activity obtained from the different saturations of ammonium sulfate had significant differences, and the most active part was seen between 40–60%, consistent with Gaunt’s study [19]. Then, the sediment that had undergone double precipitation was dissolved in 50 mM citrate-phosphate buffer with minimum volume and then dialyzed against the same buffer for more than 48 h. Thereafter, the dialyzed enzyme solution was dried by lyophilization (Christ, Alpha 1-4 LD plus, Osterode, Germany) and stored at 4 °C for use in the following experiments.

After precipitation and dialysis, enzyme activity was improved by about 20% (data not shown), and further purification was hard to conduct because of the sensitivity of the oxidase and the long reaction times. Additionally, the enzyme was stable during ammonium sulfate precipitation but had not yet been successfully purified beyond this procedure [18].

### 3.4. Reaction System and Determination of Tocopherol Oxidase Activity

Reactions were performed mainly according to the previously outlined method [18]. The reaction was conducted under magnetic stirring (Lichen, DF-101S, Shanghai, China) at 280 rpm and a temperature of 20 ± 2 °C, which was controlled by the water bath. During the reaction progress, 300 µL of the sample was removed from the incubation kettle at 2, 4, 6, and 24 h, and transferred to ethyl acetate (900 µL). Thereafter, the extract mixture was transferred to a 2 mL centrifuge tube and vortexed for 1 min, followed by 3 min centrifugation at 10,000× *g* (Eppendorf Mini Spin^®^ Plus, Hamburg, Germany). Then, the supernatant was transferred into a new tube, dried under a stream of nitrogen, and dissolved with n-hexane for further HPLC analysis. The tocopherol oxidase activity was defined as the content changes in the substrate (γ-tocopherol in this study) in units of mg/h.

### 3.5. Assay Methods of Tocopherols and Their Products

Tocopherols and their products were assayed and compared with the standard samples by using a high-performance liquid chromatographic system (HPLC-1525, Waters Corp., Milford, MA, USA) equipped with a Multi λ Fluorescence Detector, as described in the previously outlined method [24,27] with slight modifications. The tocopherol determination was performed on a Sepax HP-Silica column (5 µm, 4.6 mm × 250 mm, Sepax Technology, Inc., Newark, DE, USA) using a mixture of isopropanol and n-hexane (1.5/98.5, *v*/*v*) as the mobile phase at an isocratic elution flow rate of 0.8 mL/min. The product measurements (mainly tocopherol quinone and tocored) were performed on a C18 reverse-phase column using methanol as the mobile phase at a flow rate of 0.8 mL/min. The samples (20 μL) were injected into the HPLC system. The system was run at 295 nm and used the external standard method to determine tocopherol contents expressed in mg/kg. In detail, different contents (0.001, 0.01, 0.02, 0.04, 0.08, 0.1, and 0.2 mg/mL) of α- and γ-tocopherols were prepared to perform the external standard method and the tocopherol content was calculated.

#### Semi-Synthesis of Tocopherol Quinone (TQ) and Tocored

**Figure 7:**
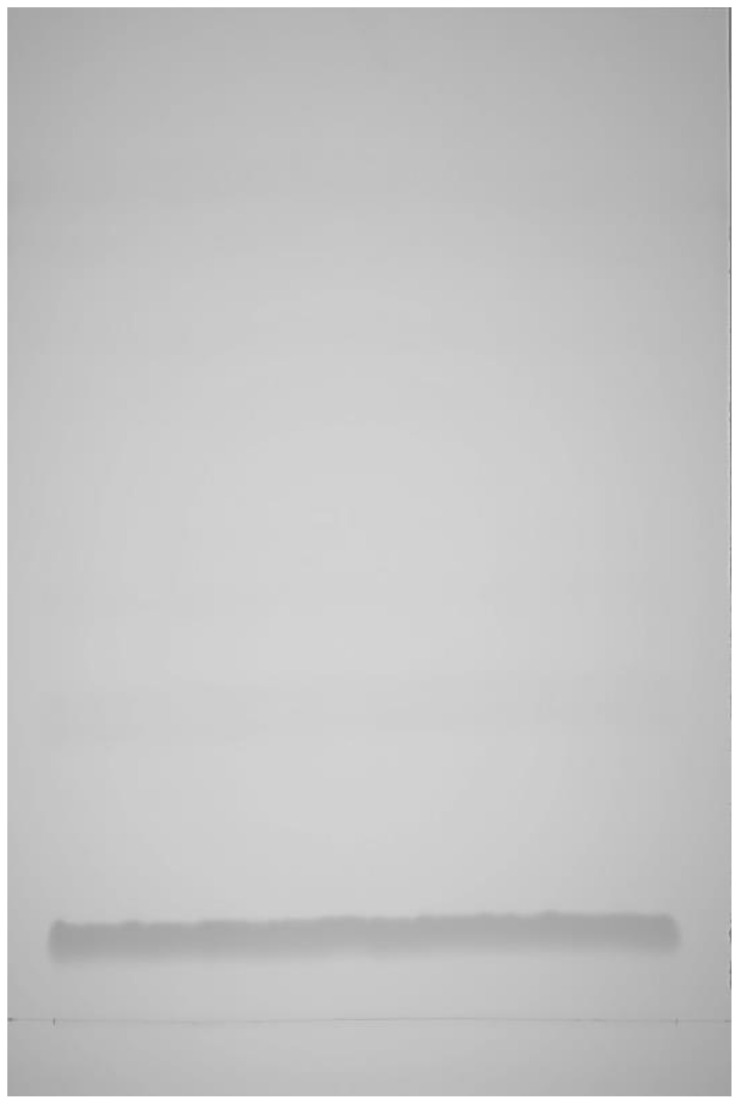
Preparative TLC chromatogram of α-TQ.

**Figure 8:**
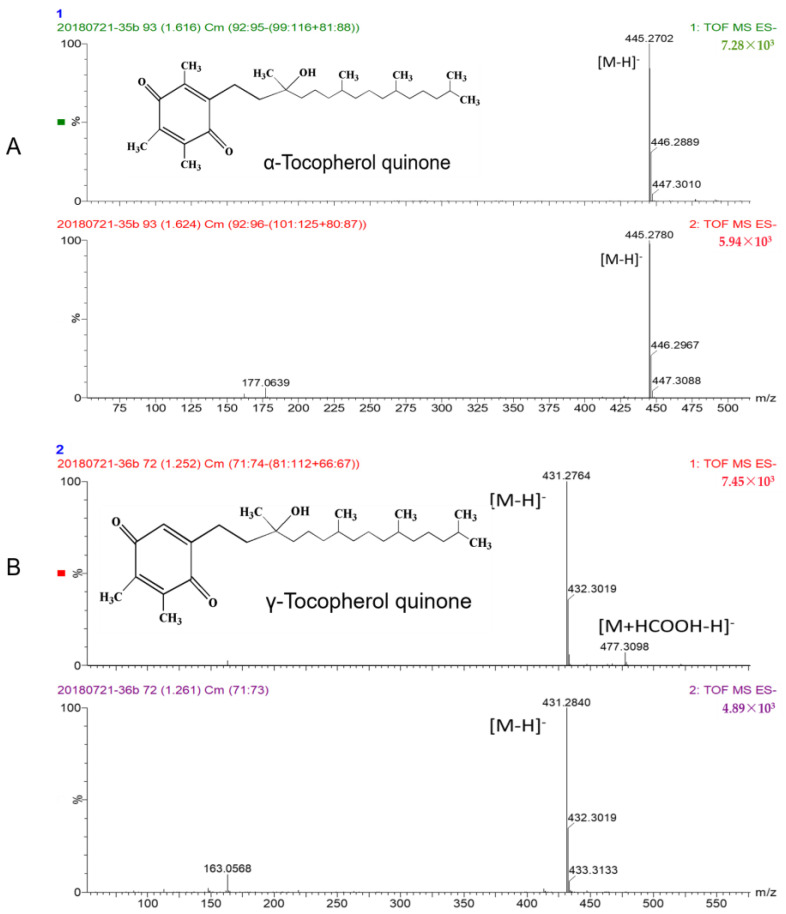
MS spectrum analysis of α-TQ (**A**) and γ-TQ (**B**).

Since there were no standards for TQ and tocored and relatively limited knowledge available on these substances, it was not easy to identify the products that the reaction produced. According to a previous study, TQs are the main products of tocopherol oxidation via enzymes [20,21], and their preparation and structures are shown in Figure 7 and Figure 8. In order to analyze the composition of the products after the reaction, the synthesis of these two compounds was first conducted, while the synthesis of TQ was carried out according to procedures published previously [31,32]. α-TQ and γ-TQ were semi-synthesized by the oxidation of parent tocopherols with hydrated FeCl_3_. Briefly, a solution of α-tocopherol in anhydrous diethyl ether (100 mg in 1 mL) was first prepared, and FeCl_3_ solution (0.2 g in 2.5 mL of methanol/water, 50/50, *v*/*v*) was added. The aqueous phase was removed after 30 min of agitation at room temperature. The remaining tocopherols in the ether phase underwent further reaction with the FeCl_3_ solution again twice, and the ether phase was then extensively washed with water (10 times) to remove FeCl_3_. The ether phase was dried by evaporation with a rotary evaporator (RE-52AA, Yarong, Shanghai, China) and dissolved in n-hexane. Similar processes for γ-TQ were conducted with less γ-tocopherol used. A preparative thin-layer chromatogram using a silica gel plate (Demision, 100 × 200 mm; thickness: 0.20–0.25 mm; particle size: 10–40 µm, Haiyang, Qingdao, China) was used to separate α-TQ and γ-TQ from other unwanted substances. A combination of n-hexane:anhydrous ether (4:1) was finally chosen to obtain the crude α-TQ (Figure 7) and γ-TQ samples. Tocored was synthesized according to the procedures we reported [27]. All tocopherols and their derivatives were stored at −20 °C before use in experiments.

The identification of α- and γ-TQ with high purities (≥92%) were determined via a liquid chromatograph mass spectrometer (LC-MS) method that had been published previously [15,27]. In detail, the products were dissolved in methanol and directly injected into the Waters ACQUITY Ultra Performance Liquid Chromatography (Waters Corporation, Milford, MA, USA), equipped with a Waters ACQUITY PDA detector and Waters MALDI SYNAPT Q-TOF–MS. The column was ACQUITY UPLC BEH C18 (50 mm × 2.1 mm id, 1.7 µm particle size) (Waters Corporation, Milford, MA, USA), with the column container set at 45 °C. The injection volume was 3 µL, and the mobile phase was methanol, with the flow rate set at 0.3 mL/min. The ion source operated in the positive ion mode for tocored and negative ion mode for TQ. The voltages of the capillary, skim, and detector were 3500, 30, and 1800 V, respectively. Other MS conditions were as follows: source block temperature, 100 °C; desolvation temperature, 400 °C; desolvation gas flow rate, 700 L/h; cone gas flow rate, 50 L/h; collision energy, 6 and 20 V; and scan range, 20–2000 *m*/*z*.

### 3.6. Effect of pH and Temperature on the Tocopherol Oxidase Stability and Activity

The functions of pH and temperature were examined using a 100 ug/mL γ-tocopherol in 50 mM citrate–phosphate buffer. The pH values of the buffer systems ranged from 3.5 to 7.5, with a 0.2 M sodium acetate–acetic acid buffer for pH 3.5–5.5 and a 0.2 M sodium phosphate buffer for pH 6.5–7.5. The optimum temperature was determined within a range of 4–60 °C at the optimum pH value.

### 3.7. Chemical Inhibition

Ascorbic acid and L-cysteine, the two main chemical inhibitors of PPO, were analyzed in this study to determine their inhibitory effect on tocopherol oxidase [18]. An inhibitor (1 mL) was added to the 9 mL reaction mixture (5 mL buffer, 2 mL substrate, and 2 mL lecithin). After incubation for 6 h, the mixture was extracted in order to assay the remaining content of the substrate by HPLC.

### 3.8. Statistical Analysis

All data were analyzed using SPSS 22.0 (SPSS Inc., Chicago, IL, USA), and the significant differences between samples were performed via a one-way ANOVA through Duncan’s multiple-range test with the statistical significance expressed as *p* < 0.05.

## 4. Conclusions

The present study reported the purification and detailed characterization of tocopherol oxidase from corn germs for the first time. This oxidase showed a specific activity with different rates towards tocopherol homologs. In the characterization test, the enzyme showed the highest catalyze activity at an optimum pH of 4.5 and temperature of 20 °C and could be completely inhibited by ascorbic acid. Tocored, which was considered to be a precursor of color reversion, was detected in the reaction mixtures, indicating that the oxidation of γ-tocopherol during the crushing of corn germs may be related to the oil color reversion.

However, due to edible oil being a complex natural mixture with numerous triacylglycerols and other minor components, a multitude of reactions may take place during the process of extraction and storage of the oil. Furthermore, this work attempted to understand the color reversion of edible oil from the enzyme perspective; however, the primary characterization of the oxidase might not be sufficient to explain the relationship between the enzyme and the color reversion. For these reasons, the catalytic mechanism and the relationship between tocopherol oxidase and the color reversion of edible oil are largely unknown and require further research to propose more effective treatments suitable for use on an industrial scale.

## Figures and Tables

**Table 1:** Effects of the two main inhibitors on the activity of tocopherol oxidase (% tocopherol inhibition).

Inhibitors	Inhibitor Concentration (mM)		
	0.2	0.5	1.0
Control	16.6 ± 2.8 ^c^	16.6 ± 2.9 ^c^	16.6 ± 2.4 ^c^
Ascorbic acid	89.6 ± 1.8 ^a^	91.7 ± 2.7 ^a^	97.4 ± 0.4 ^a^
L-cysteine	52.8 ± 4.3 ^b^	54.6 ± 3.7 ^b^	57.2 ± 3.9 ^b^

Different letters demonstrated significant differences compared to the control (*p* < 0.05).

## Data Availability

The data presented in this study are available on request from the corresponding author.

## Sample Availability

Samples of the compounds are not available from the authors.
